# First episode psychosis and the use of psychostimulants - a case report

**DOI:** 10.1192/j.eurpsy.2021.2044

**Published:** 2021-08-13

**Authors:** T. Teixeira, S. Martins, V. Carvalho

**Affiliations:** Departamento De Psiquiatria E Saúde Mental, Centro Hospitalar Tâmega e Sousa, Penafiel, Portugal

**Keywords:** First episode psychosis, attention deficit hyperactivity disorder, stimulants

## Abstract

**Introduction:**

Stimulants are considered the mainstay of treatment for attention-deficit/hyperactivity disorder (ADHD), and most patients are put on a long-term regimen with these psychostimulants. However some children treated with psychostimulants have reported psychosis as an adverse effect.

**Objectives:**

Understand the capacity of psychostimulant medications to induce psychotic symptoms and determine the frequency of such reactions in adolescents and young adults.

**Methods:**

Non-systematic review of the literature in English, through research in PubMed. Additionally, a clinical case is exposed, which was treated at the psychiatric inpatient unit of the Tamega e Sousa hospital center.

**Results:**

Some patients, including some with no identifiable risk factors, can develop drugrelated signs or symptoms of psychosis or mania, such as hallucinations, at usual doses of frequently used ADHD drugs. Age of onset of psychosis can be significantly earlier in individuals with a history of stimulant use. In our clinical case, a young man of 18 years, previously diagnosed with ADHD, was medicated with atomoxetine two months prior being admitted to our psychiatric unit. There was no reported history of a similar psychiatric condition, and no risk factors were identified. At admission, he had bizarre behavior, with allucinatory activity and delusions of persecution. Atomoxetine was suspended and started oral antipsychotic, with improvement of symptoms and stabilization of the clinical condition.

**Conclusions:**

In adolescents and young adults with ADHD undergoing stimulant therapy, any psychotic symptoms or mood changes need to be carefully assessed at regular intervals by the physicians and the caregivers, in order to observe change in the symptoms.

**Disclosure:**

No significant relationships.

